# Current and future implications of basic and translational research on amyloid-β peptide production and removal pathways

**DOI:** 10.1016/j.mcn.2015.02.016

**Published:** 2015-05

**Authors:** C. Bohm, F. Chen, J. Sevalle, S. Qamar, R. Dodd, Y. Li, G. Schmitt-Ulms, P.E. Fraser, P.H. St George-Hyslop

**Affiliations:** a Tanz Centre for Research in Neurodegenerative Diseases, Departments of Medicine, Laboratory Medicine and Pathobiology and Medical Biophysics, University of Toronto, Krembil Discovery Tower, 6th Floor-6KD417, 60 Leonard Avenue, Toronto, Ontario M5T 2S8, Canada; b Cambridge Institute for Medical Research, Wellcome Trust MRC Building, Addenbrookes Hospital, Hills Road, Cambridge CB2 0XY, UK

**Keywords:** Alzheimer, Abeta, Amyloid, Tau, Genetics, Next generation sequencing, PICALM, SORL1, EPHA1, APOE, APP, Vaccine, Secretase, Nicastrin, Presenilin, PSEN1, Neurodegeneration, Dementia

## Abstract

Inherited variants in multiple different genes are associated with increased risk for Alzheimer's disease (AD). In many of these genes, the inherited variants alter some aspect of the production or clearance of the neurotoxic amyloid β-peptide (Aβ). Thus missense, splice site or duplication mutants in the presenilin 1 (PS1), presenilin 2 (PS2) or the amyloid precursor protein (APP) genes, which alter the levels or shift the balance of Aβ produced, are associated with rare, highly penetrant autosomal dominant forms of Familial Alzheimer's Disease (FAD). Similarly, the more prevalent late-onset forms of AD are associated with both coding and non-coding variants in genes such as SORL1, PICALM and ABCA7 that affect the production and clearance of Aβ. This review summarises some of the recent molecular and structural work on the role of these genes and the proteins coded by them in the biology of Aβ. We also briefly outline how the emerging knowledge about the pathways involved in Aβ generation and clearance can be potentially targeted therapeutically. This article is part of Special Issue entitled "Neuronal Protein".

## Highlights

• Rare, medium and common variants in at least 20 genes are associated with AD.
• Multiple genes affect processing of APP and production of Aβ peptide.
• Others relate to immunity, inflammation, vesicular trafficking, and lipid metabolism.
• These genes reflect steps in a complex metabolic/signalling cascade.
• Nodal points in these pathways may represent potential therapeutic targets.

## 1 Introduction

The neuropathology of Alzheimer's disease (AD) is characterised by abnormal protein deposits. One of these protein aggregates is composed of hyperphosphorylated tau proteins, which assemble into intraneuronal neurofibrillary tangles (NFT) (Ballatore et al., 2007; Buée et al., 2000; Goedert, 2005; Lee et al., 2001; Terry, 1963). The second principal protein aggregate, composed of the amyloid β-peptide (Aβ), accumulates in the extracellular space of the brain as senile plaques (SP) and in perivascular deposits, where it gives rise to amyloid angiopathy (Glenner and Wong, 1984; Masters et al., 1985). However, the brains of AD patients often also exhibit an abnormal accumulation of other abundant proteins, including α-synuclein (in Lewy bodies) or TDP43 (Josephs et al., 2014). It is clear that the pathological accumulation of both tau and Aβ is important and necessary components of the disease process. However, the exact functional relationship between these two processes remains the topic of intense research (Vossel et al., 2010; Ittner et al., 2010). This review will focus upon the biology of the Aβ peptide.

### 1.1 Production of AΒ

**Fig. 1:**
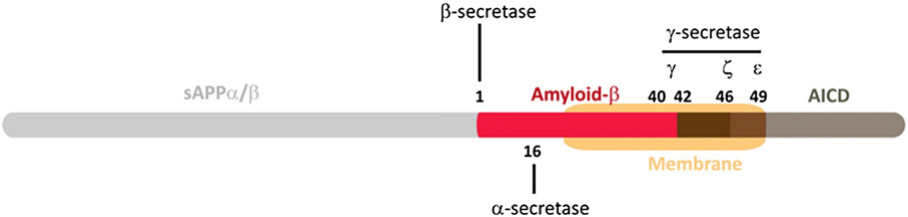
Schematic of the APP holoprotein showing the location of the transmembrane domain (orange) and the relative sites of cleavage by α-secretase, β-secretase and γ-secretase, which respectively generate: soluble sAPPα and APP-CTFα; soluble sAPPβ and APP-CTFβ; and Aβ and the amyloid intracellular domain (AICD).

**Fig. 2:**
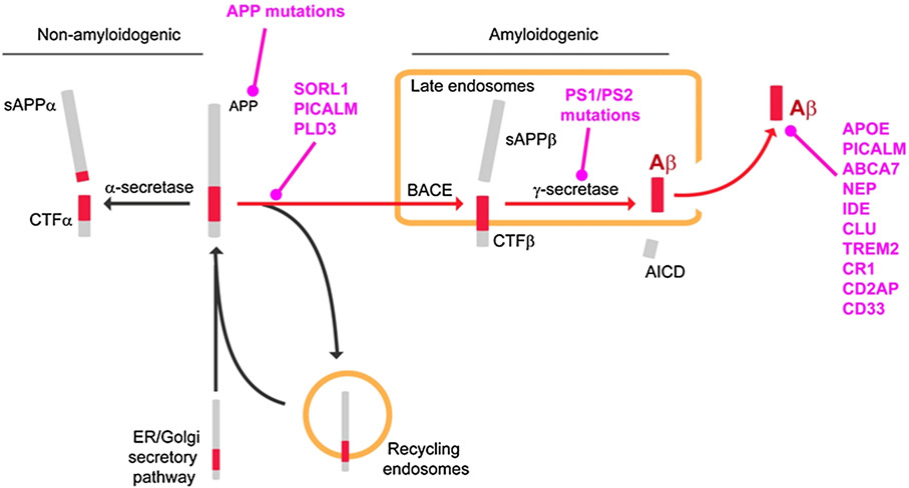
Schematic of APP processing pathways that are either non-amyloidogenic (α-secretase and recycling endosome pathways) or amyloidogenic (β-secretase and γ-secretase cleavage). The site of action of various AD-associated mutations is denoted in pink.

Aβ is a cleavage product of the amyloid precursor protein (APP). APP, a type I transmembrane protein (Figs. 1, 2), can shed its extracellular N-terminal domain (sAPP) through two independent proteolytic cleavage pathways. The enzymatic entities that lead to Aβ cleavage are broadly referred to as secretases. In the stoichiometrically predominant pathway (Ray et al., 2011), APP is initially subject to α-secretase cleavage, which can be executed by one of several members of the ADAM (a disintegrin and metalloproteinase domain) protease family, including ADAM10, ADAM17, ADAM9 and ADAM19 (Esch et al., 1990; Sisodia et al., 1990; Asai et al., 2003; Fahrenholz et al., 2000; Fuwa et al., 2007; Lammich et al., 1999). ADAM10 is the dominant α-secretase in the brain and recently two rare mutations in ADAM10 were identified which were suggested as predisposing for late-onset Alzheimer's disease (Suh et al., 2013). However, other groups have yet to confirm these findings. The α-secretase-dependent pathway is referred to as the non-amyloidogenic pathway because it cleaves the APP molecule close to the outer face of the cell membrane between residues in the centre of the Aβ peptide, thereby precluding the subsequent formation of intact Aβ peptides.

The alternative and less abundant sheddase reaction is mediated by β-secretase (BACE), which cleaves APP at the N-terminal boundary of the Aβ peptide domain, located in extracellular proximity to the plasma membrane at a site seventeen residues N-terminal to the canonical α-secretase cleavage site (Figs. 1, 2). β-Secretase cleavage requires prior re-cycling of APP from the cell surface (Haass et al., 1992; Lai et al., 1995; Vassar et al., 2014), a process which likely involves clathrin-mediated endocytosis of the full length APP by proteins such as PICALM, BIN1 and CD2AP. Following endocytosis, APP is targeted to specific subcellular compartments by intracellular vesicular protein sorting receptors. These interactions with sorting receptors, including SORL1 determine whether the full-length APP holoprotein is redirected to the retromer compartment, or allowed to drift into the late endosomal compartment (Rogaeva et al., 2007; Bhalla et al., 2012; Lane et al., 2012; Seaman, 2012; Vardarajan et al., 2012)).

The resulting membrane bound C-terminal fragments (CTF) of APP, generated by either α-secretase or by β-secretase cleavage activities, then undergo a secondary intramembrane endoproteolysis, designated γ-secretase cleavage, by a membrane-bound, multimeric protein complex, known as the presenilin complex (often also casually termed the “γ-secretase complex”). The presenilin complex is composed of four proteins (Edbauer et al., 2003): presenilin 1 (Sherrington et al., 1995) or presenilin 2 (Rogaev et al., 1995; Finckh et al., 2000), nicastrin (Yu et al., 2000), aph-1 and pen-2 (Francis et al., 2002) (see below). γ-Secretase cleavage products of APP-CTF are then extruded from the plasma membrane into the intracellular compartment (amyloid intracellular domain — AICD) or into the extracellular compartment (Aβ from the BACE pathway; p3 from the α-secretase pathway) (Figs. 1, 2).

**Fig. 3:**
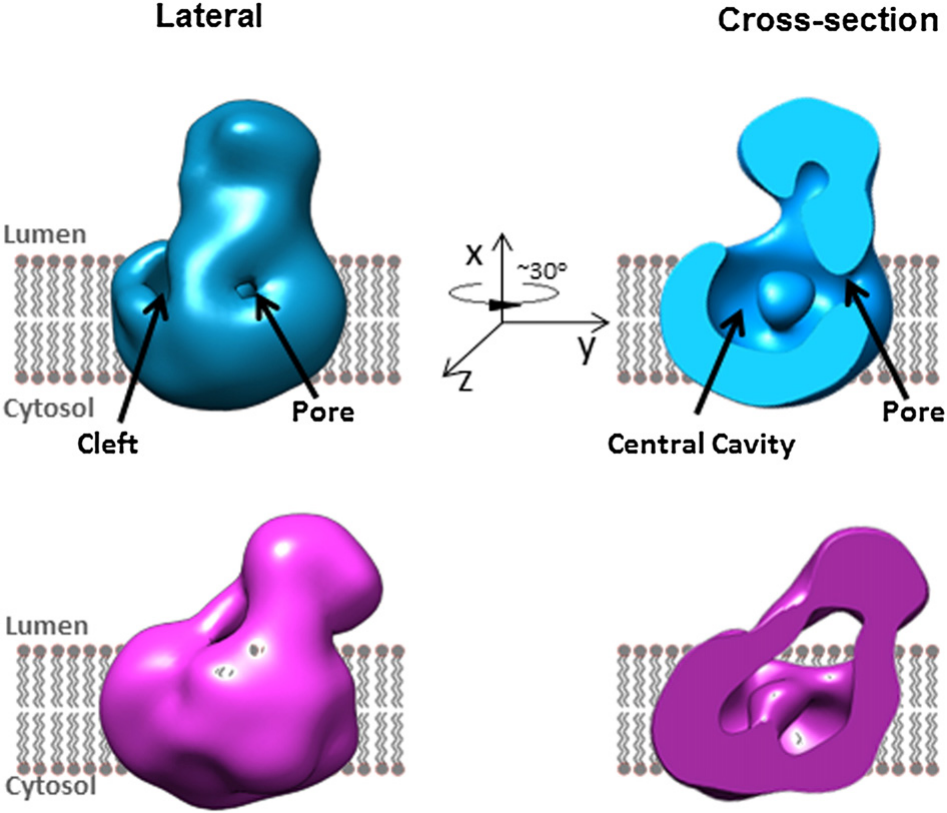
3-D rendition of the 17 Å structure of the human presenilin complex under native conditions demonstrating the presence of a soluble head domain containing the ectodomain of nicastrin and a membrane-bound body containing the transmembrane domains of the other presenilin complex component proteins (PS1/PS2, PEN-2, APH1 and nicastrin). The lower panel shows the conformational shift induced by binding of a non-transition state analogue inhibitor, which results in closure of the lateral cleft and compaction of the complex. The lateral cleft may represent an important structure, which permits access of substrates to the hydrophilic catalytic pocket protected inside the centre of the body domain.

During the last decade, molecular biological studies, and more recently, structural biology studies, using both negative stain electron microscopy and cryo-electron microscopy methods, have begun to shed light on how the presenilin complex completes the process of Aβ peptide production via a series of cleavages (termed γ, ε, ζ site cleavages) within the transmembrane domain of APP (Fig. 1). The presenilin complex recruits substrates by docking them at a putative initial substrate docking site on the surface of the complex (Kornilova et al., 2005). The substrates are then drawn into a water-accessible cavity inside the complex, allowing hydrolysis of the scissile peptide bond in the transmembrane domain (Li et al., 2014; Lu et al., 2014) (Fig. 3). Some insights into how this might be achieved have been acquired from analysing the effects of binding of peptide-like γ-secretase inhibitor compounds such as Compound E (Li et al., 2014). These studies suggest both that the structure of the complex is quite labile, and that it may exist in several different conformational states. Shifting between these different activity/conformational states is regulated by various (often reciprocal) long-range allosteric interactions (Elad et al., 2014; Li et al., 2014). For instance, we have shown that binding of substrate to the Initial Substrate Docking Site opens up a presenilin complex binding epitope for the peptidomimetic inhibitor Compound E. However, the subsequent occupancy of this peptidomimetic inhibitor binding site has a reciprocal effect on the Initial Substrate Docking Site, causing it to close. We have proposed that this represents the mechanism of a lateral gate that protects the hydrophilic catalytic site but allows intermittent access of substrate transmembrane domains. Specifically, we propose that binding of the substrate to the Initial Substrate Docking Site results in “opening up” a translocation pathway from the surface of the complex to the catalytic pocket. Subsequent occupancy of the translocation pathway (containing the Compound E binding site) causes the allosteric closure of the Initial Substrate Docking Site, allowing the substrate to access the catalytic pocket while protecting the hydrophilic catalytic pocket from the hydrophobic contents of the membrane.

Following access to the catalytic site, the complex cleaves the substrate protein through the transmembrane domain in a series of three cleavages that are presumed to occur sequentially beginning first at the ε-cleavage site close to the cytoplasmic surface of the membrane and proceeding to the γ-site in the middle of the transmembrane domain. The initial cleavage step (ε-cleavage) thus occurs 49 amino acids from the BACE cleavage site at the cytoplasmic side of the transmembrane domain (Chow et al., 2010; Fuwa et al., 2007; Haass et al., 2012; Takami et al., 2009; Tomita and Iwatsubo, 2013; Weidemann et al., 2002) Subsequently, ζ-cleavage follows at position 46 from the BACE cleavage site, releasing a small C-terminal fragment, which in all likelihood is degraded rapidly (Zhao et al., 2004). Finally, following cleavage at the γ-cleavage site, the most abundant Aβ peptide, Aβ40, is released. However, the γ-site cleavage mechanism is imprecise and Aβ peptides of varying lengths (e.g. Aβ38, Aβ42 and others) can be detected (Matsumura et al., 2014).

### 1.2 Clearance, chaperoning and degradation of Aβ

Following its production and release into the extracellular space, Aβ is cleared through several potential mechanisms. A major, and potentially the principal, clearance pathway from the brain is via transcytosis across the blood–brain barrier into the vascular lumen (Pflanzner et al., 2010). The molecular mechanisms underpinning this mode of clearance are still being worked out but may involve binding of APOE loaded with Aβ to cell-surface receptors such as LRP1 (Sagare et al., 2007; Deane et al., 2004), followed by clathrin-mediated endocytosis through molecular processes potentially involving proteins such as BIN1 and PICALM.

A second pathway for the physiological removal of Aβ is proteolytic degradation involving enzymes such as insulin degrading enzyme (Qiu et al., 1998) and neprilysin (Iwata et al., 2000; Miners et al., 2008).

Third, because of its propensity to aggregate, monomeric Aβ peptide can also assemble into soluble oligomers and then into more stable fibrils. Recent work has clearly established that the principal neurotoxic species of Aβ are the soluble oligomers, and that fibrils per se are relatively less toxic (although they serve as a source for shedding of soluble oligomers) (Knowles et al., 2014). The biophysics of Aβ assembly and compaction into neurotoxic oligomers has been extensively studied by several mechanisms, including single molecule analysis (Knowles et al., 2014), which have revealed that there are several interdependent processes which govern nucleation, oligomer formation, compaction from globular to β-sheet-rich conformation, fibril elongation and fragmentation ((Lu et al., 2013); see review (Knowles et al., 2014)). Importantly, these processes are significantly modulated by the presence of various extracellular chaperone proteins, including clusterin (CLU), which influence both the aggregation and disaggregation of Aβ by directly binding Aβ oligomers (Narayan et al., 2012, 2014a). Molecular chaperones such as cyclohexanehexol and human Brichos domains, directed at modulating Aβ aggregation, are currently being explored as potential therapeutic targets (Cohen et al., 2015; McLaurin et al., 2006).

Finally, the presence of extracellular misfolding protein aggregates such as amyloid oligomers and fibrils is known to activate innate immune responses via complement pathways, including complement receptor (CR1), and microglial signalling elements, such as TREM2 (see review) (Heneka et al., 2014; Schwartz and Shechter, 2010). While activation of innate immune responses may initially be a protective response directed at removal of these mis-folded proteins, there is increasing evidence that chronic activation of these pathways may lead to injury of neurons via the inflammasome (Heneka et al., 2013; Tan et al., 2013).

## 2 Basic science implications for the amyloid hypothesis

Following his isolation and identification of a partial amino acid sequence of Aβ peptides in the brain of AD patients, George Glenner proposed the hypothesis that AD might arise from the accumulation of misfolded β-sheet proteins in a manner analogous to systemic amyloidoses (Glenner and Wong, 1984). This hypothesis is now widely, although not universally, accepted by the field, because several subsequent observations, summarised below, have supported the notion that Aβ peptides play a central role in the pathogenesis of this disease. However, it is critical to underscore here that Aβ accumulation per se is not sufficient for the development of full-blown AD, and that tau deposition also plays an important, and perhaps essential, downstream role.

The lines of evidence supporting an important role for Aβ include the fact that a small percentage of AD cases have inherited AD in an autosomal dominant manner. Patients with familial Alzheimer's disease (FAD) carry mutations in either the APP (Goate et al., 1991), presenilin 1 (PSEN1) (Sherrington et al., 1995) or presenilin 2 genes (PSEN2) (Rogaev et al., 1995; Finckh et al., 2000) (Fig. 2). All three gene products are intimately involved in the production of Aβ, and the FAD-associated mutations in these genes all affect Aβ production and biology, albeit in subtly different ways. Some FAD mutations in PS1 (Citron et al., 1997), PS2 (Scheuner et al., 1996) and APP (Citron et al., 1992) shift Aβ production from Aβ40 to the more aggregation-prone Aβ42. Other mutations generate more Aβ overall by: 1) providing more APP substrates (e.g., APP duplications (Rovelet-Lecrux et al., 2006) and Down's syndrome (Wisniewski et al., 1985)); 2) rendering APP more accessible to BACE (e.g., the APP KM670/671NL Swedish APP mutation (Haass et al., 1995)); or 3) increasing the aggregation propensity of Aβ peptides (e.g., APP E693G Arctic mutation (Nilsberth et al., 2001)).

**Fig. 4:**
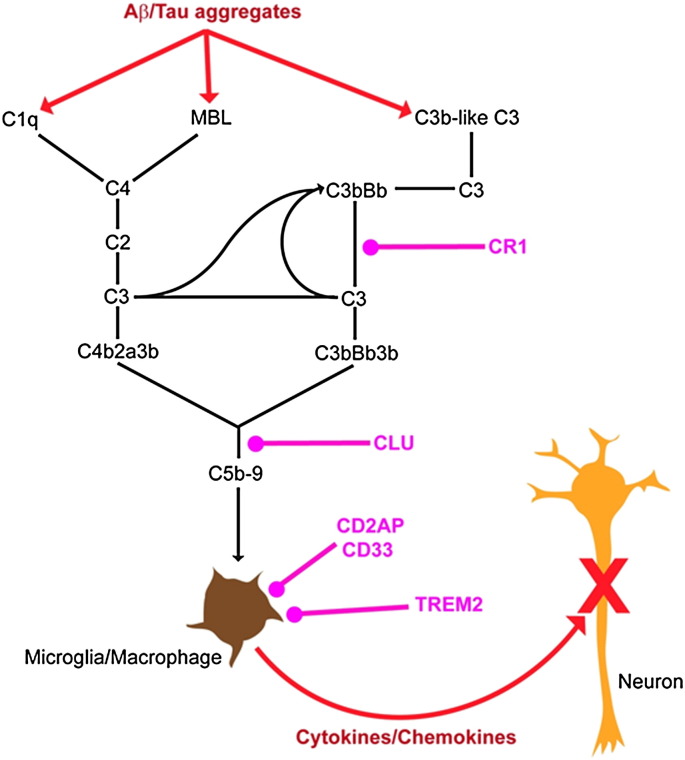
Schematic diagram of the complement cascade and location of innate immune genes associated with risk for AD.

Although the majority of monogenic autosomal dominant forms of FAD have a much earlier age of onset than observed in the more common late-onset sporadic forms of AD, both forms share very similar clinical and neuropathological features, leading to the assumption that both diseases share a molecular basis. This link has recently been further strengthened by the observation that several of the genes for late-onset AD discovered by candidate gene association studies, genome wide association studies or whole exome sequencing studies are also involved in APP processing (Figs. 2, 4). These genetic studies have now identified a long list of genes in which common or rare variants (either in non-coding regulatory sequences or in coding sequences) are associated with small increments in risk for AD (Cruchaga et al., 2014; Guerreiro et al., 2013; Harold et al., 2009; Hoglinger et al., 2011; Lambert et al., 2013; Naj et al., 2011). Bioinformatics analyses have broadly grouped these genes into functional categories marked by proteins that contribute to vesicular protein trafficking, lipid metabolism, and inflammation. Significantly, several of these genes appear to alter APP processing and Aβ production. Thus variants in SORL1, PICALM, ABCA7, ADAM10 and PLD3 have both been implicated in increasing Aβ production by altering the intracellular processing of APP, and/or been shown to modulate the uptake of already produced Aβ (Cruchaga et al., 2014; Vardarajan et al., 2015) (Figs. 2, 4).

Several other AD risk gene alleles are likely to be involved in aberrant responses to the accumulation of misfolded extracellular Aβ aggregates and, in particular, to aberrant innate immune responses to these aggregates (Fig. 4). For example, both coding sequence and non-coding sequence variants in the clusterin (CLU) gene (Bettens et al., 2012), which encodes a molecular chaperone, are associated with increased risk for late-onset AD. Although the precise effects of these AD-associated variants have not yet been defined at a molecular level, it is clear that clusterin binds Aβ oligomers and prevents further aggregation (Narayan et al., 2012). Similarly, both coding (rare sequence variants and a common in frame insertion) and non-coding sequence variants in the complement receptor 1 (CR1) have been associated with late-onset AD (Hazrati et al., 2012; Jun et al., 2010; Naj et al., 2011; Wyss-Coray et al., 2002). Again, while the molecular effects of these mutations on the clearance of Aβ aggregates and the innate immune response to them has not been investigated, it seems likely that these variants will modulate innate immune pathway activation to these aggregates (Biffi et al., 2012; Neher et al., 2014; Thambisetty et al., 2013). Alterations in microglial activity in response to the presence of Aβ aggregates are also a notable feature of the neuropathology of AD. Recently, CD33 (Bertram et al., 2008; Hollingworth et al., 2011; Naj et al., 2011), TREM2 (Guerreiro et al., 2013; Jonsson et al., 2013) and TREML2 (Benitez et al., 2014) have been associated with altered risk for late-onset AD (Fig. 4). The putative protective effect of rare missense variants in TREML2 has not yet been confirmed. CD33 failed to achieve genome-wide significance in a subsequent very large meta-analysis of 74,046 individuals (Lambert et al., 2013). These genes can potentially either directly or indirectly modulate the response of microglia to Aβ, reducing the uptake of Aβ by microglia and the subsequent activation of microglia (Griciuc et al., 2013; Kleinberger et al., 2014).

## 3 Recent studies address some of the criticism of the Aβ Cascade hypothesis

While it is clear that the presence of intraneuronal tau aggregates is an important component of the pathogenesis of AD, there have been two major criticisms of the amyloid cascade. The first criticism is that appearance of tau tangles correlates well with the disease severity (Goedert, 2005). This is not the case for senile plaques (Dickson et al., 1995) and it is not uncommon to find plaques in people who have not been diagnosed with AD in life. A potentially more substantive criticism has been based on observations from cross-sectional studies of tau pathology in subjects of varying ages. Some of these investigations have suggested that the earliest pathological change in AD may be the accumulation of tau aggregates in temporal lobe neurons, which become more prevalent and more widespread in older individuals (Braak and Braak, 1998). This observation has been interpreted to suggest that the first change in AD is the accumulation of tau aggregates, which subsequently spread across the brain.

One of the translational products of the work on the amyloid cascade has been the development of CSF and neuroimaging biomarkers for the measurement of Aβ and tau accumulation in the brain of asymptomatic and symptomatic (Jack and Holtzman, 2013; Lleo et al., 2015; Risacher and Saykin, 2013; Thal et al., 2014; Wicklund and Petersen, 2013). These tools have enabled correlative cognitive, neuroimaging and neuropathological studies which have begun to suggest an alternative interpretation. Specifically there is emerging evidence to support the view that these apparently disparate observations actually reflect the presence of two different processes: 1) age-related neurodegeneration in the hippocampus with tauopathy but without Aβ pathology (primary age-related tauopathy — PART); and 2) independent Aβ-associated disease which begins with the early preclinical deposition of Aβ in the neocortex followed by subsequent tau accumulation, inflammation and cognitive impairment, as would be predicted by the amyloid cascade hypothesis (Crary et al., 2014; Jack, 2014; Jack et al., 2014).

## 4 Alzheimer's disease therapies targeting Aβ

The molecular genetic, molecular biological, cell biological and animal modelling work summarised above, has clearly identified several metabolic and signalling pathways that result in the accumulation of Aβ peptides, hyperphosphorylation and intraneuronal aggregation of tau, and the activation of innate immune and inflammatory pathways. As the molecular details of the nodal control points within these pathways become known in greater detail, it is likely that systems biology approaches will be able to mine useful therapeutic targets from these pathways (Rhinn et al., 2013; Santiago and Potashkin, 2014; Zhang et al., 2013). Indeed, the literature is replete with in vitro, cellular and animal modelling studies of candidate therapeutics within these pathways (see reviews (Hampel et al., 2014; Huang and Mucke, 2012)). An in-depth review of this area of emerging work is beyond the scope of the present review. We will however briefly point out several components of the Aβ production and clearance pathways which are currently being investigated as therapeutic targets (Narayan et al., 2014b).

One of the best studied strategies for prevention and/or treatment of AD has been to attempt to reduce Aβ production either by augmenting α-secretase activity, by decreasing BACE activity or by inhibiting γ-secretase activity. Augmenting α-secretase activity has been experimentally induced by activation of 5-hydroxytryptamine 4 (5-HT4) receptors (Pimenova et al., 2014), or by overexpression of matrix metalloproteinase 9 (Fragkouli et al., 2014) or melatonin (Shukla et al., 2015). Attempts to inhibit BACE activity were initially frustrated by the large catalytic pocket of BACE and by the need to have compounds with good penetration of the blood–brain barrier. There exist continuing concerns about the potential effect of partial inhibition of other BACE substrates such as neuregulin (Willem et al., 2006) or subunits of voltage-gated sodium channels (Kim et al., 2007). However, at least one highly effective BACE inhibitor, which induces substantial and prolonged suppression of Aβ production in human brain, is now in early clinical trials (Butini et al., 2013; Vassar et al., 2014; Yan and Vassar, 2014). Similarly, much attention has been paid to the development of inhibitors/modulators of γ-secretase. Such compounds were readily identified but typically suffered from mechanism-based toxicity arising from suppression of other signalling pathways dependent upon γ-secretase cleavage of non-APP receptors (e.g., Notch) (Cummings, 2010; Doody et al., 2013). As a result, recent work has focused upon a heterogeneous class of compounds termed γ-secretase modulators (GSM) (reviewed in Golde et al., 2013; Hall and Patel, 2014; Pettersson et al., 2013; Crump et al., 2013). These compounds appear to act via altering the preferred γ-secretase site, and thereby down-regulate production of the more amyloidogenic Aβ42 species in favour of the less amyloidogenic Aβ38 species. These compounds do not appear to affect ε-cleavage activity. Their molecular mechanisms are not fully understood, but appear to involve long-range allosteric effects of these compounds from non-catalytic binding sites on the complex or substrate itself. In a recent study a GSM induces a conformational change of the catalytic center by binding to presenilin's luminal loop (Takeo et al., 2014). Further work is required to establish how universal this proposed mechanism is across chemically divergent GSM groups. An alternate approach to reducing the production of Aβ is to alter APP processing in a way that discourages trafficking of APP into the late endosomal pathway for β- and then γ-secretase cleavage. A promising strategy that has currently been tested in preclinical cellular models is the use of small molecule chaperones to improve retromer stability (Mecozzi et al., 2014).

The observation that oligomeric forms of Aβ appear to be the primary toxic species has led to attempts to inhibit Aβ aggregation using a variety of small molecules. One such compound, scyllo-inositol, is able to prevent the oligomerization of Aβ and leads to an improvement of both cognition and neuropathology in an AD mouse model (McLaurin et al., 2006). However, a phase 2 clinical trial based on scyllo-inositol had to be discontinued due to toxicity (Salloway et al., 2011). The therapeutic efficacy of this strategy therefore currently remains unresolved.

Several therapeutic targets have been identified in pathways involved in Aβ removal (see review, (Saito and Ihara, 2014)). Up-regulation of the putative Aβ degrading enzymes (insulin degrading enzyme, angiotensin-converting enzyme and neprilysin) have been proposed, however, it is important to bear in mind that these enzymes have multiple other activities (e.g., vasoconstriction), and dysregulation of these other activities may have mechanism-based side effects (Miners et al., 2014).

Modulation of transcytosis of Aβ by targeting LRP1 or the receptor for advanced glycation end products (RAGE) has also been proposed as a therapeutic target to modulate Aβ levels. RAGE is increased in brain blood vessels in AD cases (Yan et al., 1996). RAGE partakes in Aβ transcytosis but mediates the influx into the brain (Deane et al., 2003). RAGE inhibitors have therefore been proposed as a way to limit re-uptake of Aβ. A small molecule inhibitor of the RAGE receptor (TTP488) is currently in human phase II and III clinical trials (Saito and Ihara, 2014). The lipoprotein receptor LRP1 is important for the transcytotic removal of Aβ across the blood–brain barrier (BBB). Aβ can bind LRP1 directly or via APOE (Wisniewski and Frangione, 1992; Uchihara et al., 1995). Although APOE's interaction with Aβ has come under some scrutiny recently, multiple groups found that APOE may compete with Aβ for LRP, rather than play the role of courier (Sagare et al., 2013; Verghese et al., 2013). Expression of APOE is controlled by liver X receptor (LXR) in a heterodimer with other nuclear receptors, including retinoid X receptor (RXR) and peroxisome proliferator-activated receptor γ (PPARγ) (Laffitte et al., 2001). In 2012, a report gained wide attention describing efficient clearance of Aβ deposits in an AD mouse model after injections with bexarotene, an RXR agonist approved for cancer therapy (Cramer et al., 2012). Unfortunately, multiple groups were unable to replicate the original findings (Price et al., 2013; Tesseur et al., 2013; Veeraraghavalu et al., 2013).

Perhaps the most widely tested therapeutic approach to Aβ accumulation has been the use of active or passive immunisation therapies. Early studies in transgenic mouse models expressing mutant human APP strongly suggested that anti-Aβ antibodies were capable of disassembling the amyloid pathology and improving cognitive function (Janus et al., 2000; Schenk et al., 1999). An early trial with the active immunisation compound AN-1792 (Elan Pharmaceuticals) was halted after a subset of participants developed encephalitis (Orgogozo et al., 2003). In a follow-up study the immunised group exhibited some reduction in brain Aβ load compared to the control group but failed to improve cognitive markers in the small sample studied (Holmes et al., 2008; reviewed in St George-Hyslop and Morris, 2008). Subsequent studies based on passive immunisation with recombinant anti-Aβ antibodies have all failed to produce convincing evidence for efficacy, at least under the conditions studied in these trials, even though some of them (e.g., gantenerumab–Roche) had shown promise in a phase 2 trial (Ostrowitzki et al., 2012). Solanezumab, though generally negative, showed significant but small improvements on cognition in a subgroup of patients with mild AD (Doody et al., 2014) and because of the compound's good safety profile was selected for further prevention studies.

The reason for the almost universal absence of demonstrable efficacy of anti-Aβ immunotherapy in AD (at least as tested) is not immediately apparent. Several possible explanations have been proposed (reviewed in (Hampel et al., 2014; Spencer and Masliah, 2014)). For instance, it is conceivable that the very low penetrance of these antibodies to the brain and or the relatively modest affinity of some of these antibodies may have been insufficient to produce adequate target engagement. Alternatively, the antibodies may not have been directed to the relevant neurotoxic soluble amyloid oligomer species. Finally, it is increasingly likely that they may have been given too late in the disease course to have had an impact. Data emerging from large-scale neuroimaging initiatives, and from the longitudinal assessment of presymptomatic carriers of presenilin or APP mutations have revealed that the amyloid pathology begins more than a decade before onset of symptoms, and is followed by tau misprocessing and activation of inflammatory processes (Bateman et al., 2012; Lippa et al., 1998).

This possibility has energised attempts to define novel, sensitive biomarkers of the accumulation of Aβ and activation of downstream biochemical events for use in the preclinical detection of cases who might then be included in secondary prevention trials.

## 5 Conclusions

There is now an abundance of information arising from molecular genetic, molecular biological, cellular biological, systems biology, and structural biology which very strongly supports the notion initially espoused by George Glenner that the accumulation of aggregates of Aβ is an important and early event in the pathogenesis of both the rare autosomal dominant forms of Alzheimer's disease and also the more common “sporadic” late-onset forms of this disorder.

The knowledge of the systems biology of the pathways involved has been hugely facilitated by genetic studies of both early- and late-onset forms of this disease. These genetic studies have clearly depicted the importance of Aβ and APP, and more recently highlighted a key role for dysfunctional innate immune responses, presumably, to the presence of these neurotoxic protein aggregates.

The knowledge of the molecular and cellular biology of these diseases has encouraged the development of tractable biochemical and neuroimaging biomarkers that have underscored the observation that defects in Aβ processing occur decades before the onset of detectable clinical features. These same studies are beginning to resolve the apparently discordant observations that there is an increasing amount of tau pathology in the brains of normal elderly individuals, which becomes associated with cognitive impairment as it progresses. An increasingly likely explanation for these observations is that they represent a progressive age-related tauopathy that is probably a separate (but quite prevalent) disorder from Alzheimer's disease.

There however remain numerous important questions, particularly about the systems biology of neurodegeneration in Alzheimer's disease.

Notable amongst these questions is the unclear relationship between Aβ pathology and the equally neurotoxic tau pathology.

A second critical outstanding question that will need further work, is to identify additional details of the molecular, metabolic and signalling pathways that regulate Aβ production and toxicity, tau hyper-phosphorylation, mislocalisation and toxicity, and neuroinflammation. The cataloguing of the molecular components of these pathways, and in particular, the identification of the key nodal regulators of these pathways, will be needed in order to develop tractable therapeutic targets that inhibit the disease process but also have minimal mechanism-based toxicity. The information required to develop this comprehensive systems biology approach will likely be generated both by a continuation of existing hypothesis-driven strategies, but also by taking advantage of hypothesis-free analyses of large datasets that encompass genetics, genomics, epigenetics, proteomics, metabolomics, and exposomics.

There are also key clinical biological questions which need to be addressed.

Specifically, what is the basis for the apparent increased vulnerability of hippocampal, temporal and parietal neurons to the pathology of AD? Answers to this question may facilitate the development of sensitive assays of dysfunction in these neural circuits, which can be deployed as theragnostic markers.

A related clinical biological question that also remains unresolved is the basis for the current apparent failure of anti-Aβ therapies (at least when given during the mid-late clinical stages of the disease). One plausible explanation may be that there are critical time windows during which inhibiting Aβ accumulation and toxicity might prevent activation of downstream pathways. If these other pathways are self-sustaining once activated, then the removal of Aβ might no longer be sufficient to halt progression of the disease.

Answers to these remaining questions will require a sustained broad-based approach to research on AD at basic, translational and clinical levels for the foreseeable future.

## Author Contributions

All authors contributed to the writing of this manuscript. CB and PHStGH designed the overall approach of the review. All other authors commented on and further improved the manuscript.
